# Editorial: Acetic acid bacteria

**DOI:** 10.3389/fmicb.2023.1142659

**Published:** 2023-01-31

**Authors:** Isidoro Garcia-Garcia, Maria Gullo, Fusheng Chen, Teresa Garcia-Martinez

**Affiliations:** ^1^Department of Inorganic Chemistry and Chemical Engineering, Agrifood Campus of International Excellence ceiA3, Chemical Institute for Energy and Environment (iQUEMA), University of Córdoba, Córdoba, Spain; ^2^Department of Life Sciences, University of Modena and Reggio Emilia, Reggio Emilia, Italy; ^3^Food Biotechnology and Food Safety Laboratory, College of Food Science and Technology, Huazhong Agriculture University, Wuhan, China; ^4^Department of Agricultural Chemistry, Edaphology and Microbiology, Agrifood Campus of International Excellence ceiA3, University of Córdoba, Córdoba, Spain

**Keywords:** acetic acid bacteria, oxidative fermentation, vinegar, omics, exopolysaccharides, taxonomy, acid resistance, genetic improvement

Acetic acid bacteria (ABB) are strictly aerobic organisms that can be found in a wide variety of natural and industrial environments. Their versatility and metabolic adaptability make them microorganisms of high interest to study the optimization of obtaining their multiple products and the essential mechanisms that allow them to grow under extreme conditions. Their metabolism, particularly the role of membrane-bound and soluble dehydrogenases, may offer new opportunities in the development of innovative processes based on their capability for carrying out the incomplete oxidation of several substrates. On the other hand, the resistance of AAB to some extreme conditions, i.e., low pH values, and adaptability to many different habitats make them highly competitive bacteria; so, their interaction with other organisms and plants is a very important topic to be studied. The ability of AAB in producing exopolysaccharides is also of great interest for both research and industrial purposes. They are considered as model organisms for understanding the mechanisms of cellulose synthesis and they are until now the most efficient organisms for producing it, under controlled conditions. Finally, the current state of omic technologies and efficient genetic modification methods can be applied for a greater understanding of the physiological behavior, the recovery of new strains and/or those occurring in complex environments as well as to exploit the full potential of AAB for oxidative bioconversions.

Taking all of the above into account, this Research Topic aims to address some of the most recent advances and challenges that arise around AAB, considering some of the main areas of study: taxonomy, physiology, products and processes, microbiota analysis, omic aspects, adaptation and genetic modification and others.

Considering the taxonomic revisions and the recent advances, which include a progressive increase of species as well as the description of new genera, especially within genera that are today recognized of great biotechnological relevance, a systematic of AAB is a hot topic. From the taxonomic point of view, until 2021, 19 genera of AAB have been reported (He et al.).

The high versatility of AAB in performing microbial bioconversion is industrially exploited mainly in foods and beverages, biomedical, pharmaceutical, cosmetics and agronomical fields. Some of the main products obtained by AAB, resulting from the activity of cytoplasmic membrane-bound dehydrogenases, are sorbose, dihydroxyacetone, miglitol and acetic, gluconic, gulonic and galactonic acids; up to now, at least 86 products of this type have been reported in the literature (see Figure 1, He et al.).

**Figure 1:**
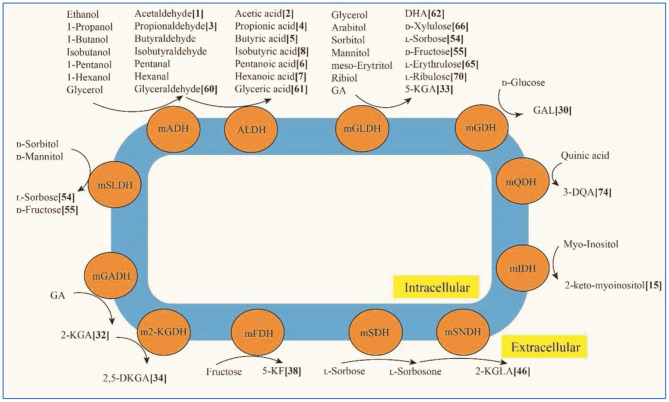
Figure 3 in He et al.. Typical membrane-binding dehydrogenases in acetic acid bacteria and their main oxidative fermentation products. mADH: membrane-binding alcohol dehydrogenase; mALDH: membrane-binding acetaldehyde dehydrogenase; mGADH: membrane-binding gluconate dehydrogenase; m2-KGDH: membrane-binding 2-keto-D-gluconate dehydrogenase; mFDH: membrane-binding D-fructose dehydrogenase; mSLDH: membrane-binding D-sorbitol dehydrogenase; mGDH: membrane-binding glucose dehydrogenase; mGLDH: membrane-binding glycerol dehydrogenase; mQDH: membrane-binding quinic acid dehydrogenasw; mIDH: membranebinding inositol dehydrogenase; mSDH: membrane-binding sorbose dehydrogenase; mSNDH: membrane-binding sorbone dehydrogenase; GA: gluconic acid; 2-KGA: 2-keto-Dgluconic acid; 2, 5-DKGA: 2, 5-diketo-D-gluconic acid; 5-KF: 5-keto-D-fructose; DHA: dihydroxyacetone; 3-QDA: 3-dehydroquinic acid; 2-KGLA: 2-keto-l-gulonic acid.

AAB, traditionally known for their ability to incompletely oxidize many sugars and alcohols, appear as the responsible organisms for vinegar production. Vinegar is, probably, one of the best known products of AAB and one of the most economically important. For this reason, and because many aspects remain still unknown, it is not surprising that there is an important field of work on it. Next references, published in this Research Topic, show some examples of topics under research on vinegar: Mizzi J et al.; Qian et al.; Román-Camacho, Mauricio et al.; Román-Camacho, García-García et al.; Xia et al.; Yang et al.

Mizzi et al. study the isolation, identification and fermentation performance of indigenous AAB from local raw materials.

On the other hand, Qian et al. are studying another of the problems that affect the behavior of the complex microbiota responsible for acetification in the production of vinegar; specifically, the interactions between prophages and the microbiota itself. The study evaluates the co-evolution of prophages and the genus *Acetobacter*; evidencing the important influence it has on the stability of the bacterial genome and thus affecting the industrial production of vinegar.

Using omic techniques, Román-Camacho, Mauricio et al.; Román-Camacho, García-García et al. approach the difficult problem of identifying and characterizing the complex microbial communities responsible for the production of vinegar, as well as the influence of the use of various culture media. Both through metaproteomics and metagenomics recognize that the microbial composition during industrial vinegar production include mainly *Komagataeibacter* members, especially *Komagataeibacter europaeus* strains. Likewise, the works show the ability of bacteria to adapt to different culture media through metabolic versatility.

Many of the products obtained by AAB are the result of complex microbial communities, either formed by AAB exclusively, or by AAB and other microorganisms. For example, the cereal vinegars produced in China are one more example of these complex ecosystems. The work of Xia et al. studies the interactions between AAB and lactic acid bacteria (LAB) during the solid-state fermentation of Shanxi aged vinegar. In this same context of complex microbial communities, Bouchez et al. carried out a review of the role of AAB in the production of sour beers. The identification, activities and, especially, the interactions at the molecular level between all microorganisms, is one of the problems in which these bacteria are almost always involved.

Another aspect that also receives a lot of attention is the one related to the study of how AAB resist the aggressive environments in which they are normally found, for example, the high acidity values during the industrial production of vinegar. Using RNA-Seq transcriptomic analysis, Yang et al. study gene regulation changes to find possible relationships with the acidity of the medium.

As previously mentioned, there are multiple products that can be obtained by AAB, for example, new sweeteners such as 5-keto-D-fructose (5-KF) from the oxidation of fructose (see Figure 1). Although it is important to know all the aspects of its synthesis, it is also important to evaluate the properties of all these products from the point of view of the final use that is intended to be made of them. In this case, in the context of the search for new, healthier sweeteners, the work presented by Hövels et al. reveals the cytotoxic potential of 5-KF for both eukaryotic and prokaryotic cells. Therefore, its use in the food sector is not recommended.

Another of the products that can be obtained with AAB and with great potential for various applications is bacterial cellulose. Anguluri et al. carry out a study on *Komagataeibacter xylinus* K2G30 to evaluate its adaptation capacity on mannitol and even on D-fructose. The strain used shows a great adaptability for the production of cellulose. All this reveals the metabolic versatility of *K. xylinus* K2G30, which suggests the possibility of developing new strategies to produce bacterial cellulose without the need to use other approaches through genetic engineering.

As already mentioned, the most important product obtained by AAB is acetic acid in the context of vinegar production, and in this sense, other microorganisms cannot compete with them. However, if we consider the possibility of using AAB for the synthesis of acetic acid for producing it as a pure commodity they cannot compete with the chemical synthesis. For this reason, Krusong et al., selected a *Paenibacillus azoreducens* strain, which presents some metabolic advantages over *Acetobacter pasteurianus* (UMCC 2951) and which, in combination with AAB, might be used for the production of acetic acid as a non-food grade commodity.

New and highly efficient methods of genetic modification can further expand the potential of this group of bacteria. In this context, Fricke et al., study the use of the L-rhamnose regulator and its target promoters from *Escherichia coli* to act on the repression or activation of certain genes in *Gluconobacter oxydans*; all this is highly interesting to achieve the redirection of carbon flows in the context of metabolic engineering. On the other hand, another extremely interesting strategy to deduce the carbon flux is through a genomic analysis using an *in-silico* approach, which allows the simulation of reconstructing genome-scale metabolic models (GEMs). An example of this can be found in Pelicaen et al., in the context of the complex fermentation of cocoa where there are species, *Acetobacter ghanensis* and *Acetobacter senegalensis*, over which others clearly prevail, *Acetobacter pasteurianus*. In this way, it is possible to deepen the knowledge of bacterial metabolism and, thus, its adaptation to different substrates and conditions and some progress could be made in many aspects, for instance, in the preparation of optimal starter cultures for this fermentation.

## Author contributions

All authors listed have made a substantial, direct, and intellectual contribution to the work and approved it for publication.

